# Residents’ perceptions of simulation as a clinical learning approach

**Published:** 2017-02-24

**Authors:** Catharine M. Walsh, Ankit Garg, Stella L. Ng, Fenny Goyal, Samir C. Grover

**Affiliations:** 1 The Wilson Centre, Ontario, Canada; 2 Centre for Ambulatory Care Education, Women’s College Hospital, Ontario, Canada; 3 Hospital for Sick Children, Ontario, Canada; 4 St. Michael’s Hospital, Ontario, Canada; 5 Centre for Faculty Development, Ontario, Canada; 6 Department of Paediatrics, University of Toronto, Ontario, Canada; 7 Department of Medicine, University of Toronto, Ontario, Canada; 8 Department of Speech-Language Pathology, University of Toronto, Ontario, Canada; 9 McMaster University, Ontario, Canada

## Abstract

**Background:**

Simulation is increasingly being integrated into medical education; however, there is little research into trainees’ perceptions of this learning modality. We elicited trainees’ perceptions of simulation-based learning, to inform how simulation is developed and applied to support training.

**Methods:**

We conducted an instrumental qualitative case study entailing 36 semi-structured one-hour interviews with 12 residents enrolled in an introductory simulation-based course. Trainees were interviewed at three time points: pre-course, post-course, and 4–6 weeks later. Interview transcripts were analyzed using a qualitative descriptive analytic approach.

**Results:**

Residents’ perceptions of simulation included: 1) simulation serves pragmatic purposes; 2) simulation provides a safe space; 3) simulation presents perils and pitfalls; and 4) optimal design for simulation: integration and tension. Key findings included residents’ markedly narrow perception of simulation’s capacity to support non-technical skills development or its use beyond introductory learning.

**Conclusion:**

Trainees’ learning expectations of simulation were restricted. Educators should critically attend to the way they present simulation to learners as, based on theories of problem-framing, trainees’ *a priori* perceptions may delimit the focus of their learning experiences. If they view simulation as merely a replica of real cases for the purpose of practicing basic skills, they may fail to benefit from the full scope of learning opportunities afforded by simulation.

## Introduction

Health professions education continues to embrace simulation to augment clinical experiences, target skills and behaviours that are difficult to acquire through traditional training, and reduce patient risk.^1^,^2^ Simulation has been defined broadly as an instructional technique that substitutes or amplifies real patient encounters with modalities such as artificial models or standardized patients that evoke or replicate substantial aspects of the real world in an interactive manner.^3^ Simulation is attractive to educators as it supports curriculum standardization and a learner-centered educational experience that enables trainees to safely commit and learn from errors.^1^ Mounting evidence regarding simulation’s effectiveness adds further validity to its integration into health professions education.^4^

Although educators are placing increasing reliance on simulation-based training and researchers continue to advance the field on substantive and methodological grounds,^4^ studies to date have largely focused on quantifying the effectiveness and impact of simulation-based education. The literature examining trainees’ perceptions of simulation as an educational methodology remains limited. To date, research evaluating learners’ perceptions of simulation has largely included survey-based studies^5^–^7^ or qualitative evaluation of *specific* simulation modalities, such as whole-body mannequins,^8^ strategies, such as just-in-time training^9^ or team training^10^ and tasks, such as catheterization.^11^ Research indicates that trainees value simulation and appreciate the simulation-based learning environment as a safe arena for training that facilitates learning without affecting patient safety.^10^,^12^,^13^ Additionally, the current literature highlights trainees’ perceptions of the importance of direct observation and feedback, realism of the simulation experience, and simulation as a means to instill confidence.^5^–^14^ While learners’ experiences are represented in the current literature, studies to date have focused narrowly on specific contexts and/or components of simulation-based training and have failed to provide a rich, nuanced understanding of learners’ perceptions of *simulation*, as a clinical learning approach. Additionally, trainees’ perceptions of the negative affordances of simulation have not be explored.

Social cognitive theory views education as a learner-centered, social, and collective process influenced by learners’ active engagement.^15^ A prominent discourse in medical education asserts that trainees are active participants in their education and their learning is influenced by their goals, attitudes, values, and experience.^16^ Consideration of a learner-centered perspective is fundamental to the education development process to help ensure active engagement and common ground; that is, educators should aim to create a shared or compatible objective between learners’ and the educational activity’s intent.^17^ Aligning with social cognitive theory, Kneebone^18^ has argued that educators need to understand trainees’ perspectives because the views of the experts responsible for designing simulation-based training may be vastly different from learners’ views. If learners view their training as unengaging, irrelevant, or if they perceive the purpose for a training approach differently (e.g., more narrowly) than the actual purpose, it may impact learning in unintended, and potentially negative, ways. Thus, a critical gap remains: a richer, more nuanced understanding of the learners’ perspective is required to help simulation educators recognize and address the needs of learners to promote active engagement and develop a shared sense of purpose. Filling this gap may help maximize the utility of simulation-based education, identify and rectify potential barriers to and unintended negative consequences^19^ of simulation-based training, guide thoughtful curricular integration, and inform future research. To address this gap, we aimed to gain a rich understanding of trainees’ perceived benefits and challenges of learning through simulation.

## Methods

An instrumental qualitative case study design was employed.^20^,^21^ Qualitative case studies are appropriate when seeking to enhance understanding of a phenomenon or topic of interest, in order to improve and inform future practices.^22^ In instrumental case study design, the “case” is set up as a bounded unit of analysis – bounded by time, place, activity, or types of people – that serves as a rich example, and therefore, an opportunity to understand the larger phenomenon of interest.^21^,^23^ In this study, the phenomenon of interest was residents’ perspectives on simulation and the chosen case is described next.

### Instrumental case description

Our case was an introductory simulation-based gastrointestinal endoscopy training course which is run annually at an academic hospital simulation centre for all incoming gastroenterology residents and second-year surgical residents to provide them with endoscopy exposure prior to their first clinical endoscopy rotation. The course, which is a required element of their curriculum, is designed to teach technical and broader non-technical competencies in colonoscopy such as decision making, communication, and leadership.^24^ One week prior to the course, residents received a course manual that explicitly outlined the course goals, relating to both technical and non-technical aspects of endoscopy, details of the course structure, and simulation-based training. Aside from the course manual, participants were not required to complete any pre-reading prior to the course. Training, which was distributed over a week, was comprised of six hours of interactive small-group lectures led by a faculty gastroenterologist that covered the theory of colonoscopy and mechanics of performance of colonoscopic procedures, interlaced with eight hours of supervised one-on-one endoscopy training on the EndoVR virtual reality simulator (CAE Healthcare Canada, Montreal, Quebec, Canada). The simulator models navigation through a colon, using a specialized endoscope that is inserted into a computer-based module with a screen showing the colon of a virtual patient. It has several standardized colonoscopy cases of varying complexity that provide users with visual and tactile feedback related to the procedure. Trainees worked through a specified list of cases on the simulator and received one-on-one guidance from an experienced endoscopist who demonstrated procedural elements of colonoscopy, answered questions, and provided individualized performance feedback as required. Subsequently, participants completed an integrated procedural performance instrument (IPPI)^25^,^26^ team training scenario with a standardized patient (human patient actors trained to simulate patients in a standard manner) and nurse to help address broader non-technical competencies related to the procedure such as situational awareness, teamwork, and communication.^24^ During the scenario pre-brief, trainees were told to perform the procedure as though they were in the real clinical setting. They were provided with a clinical scenario and asked to explain the procedure and obtain procedural consent from the standardized patient. Trainees then performed the procedure on the virtual reality simulator while interacting with the standardized patient and nurse. Following the procedure, they were expected to discuss the findings and follow-up plan with the patient. Faculty gastroenterologists (n = 8) attended a one-hour orientation session conducted by the course directors where they were instructed to provide formative feedback, intended to modify learners’ thinking and/or behavior for the purpose of improving learning,^27^ on both technical and non-technical components of performance throughout the course. To help promote a safe and supportive learning environment, residents were provided ongoing formative feedback. In addition, their performance was not formally assessed and no grades were assigned at the end of this short endoscopy training course.

### Interviews

Twelve trainees participated in three individual, semi-structured interviews (pre-course, post-course, and 4–6 weeks later) for a total of 36 interviews. The interviews lasted up to one-hour and were conducted by a single student-researcher (FG) with experience in qualitative interviewing. An additional three trainees consented, however, interviews were not conducted due to scheduling conflicts. This study received ethical approval from the St. Michael’s Hospital and Toronto Academic Health Sciences Network research ethics boards and written informed consent was obtained from all participants.

Considering the in-depth imperative of case study research, and the iterative nature of the qualitative descriptive analytic approach,^20^ multiple semi-structured interviews with each trainee were conducted, enabling the research team to gain an increasingly deeper understanding of trainees’ perceptions of simulation, both situated within, but also referenced beyond, the context of this specific course. Each resident was interviewed at three time points: immediately before the course, post-course, and 4–6 weeks later after performing colonoscopy in the clinical setting. The interviews were semi-structured and aimed at eliciting residents’ perceptions of simulation, in general, as a learning modality. During the first interview, which was conducted prior to the course, trainees were instructed to think broadly about all their experiences with simulation to date (i.e., during medical school and residency). The second and third interviews were conducted to further elucidate their perceptions, both within (the second interview) and beyond (the third interview) the context of this specific course. The third interview occurred a sufficient amount of time after the course to ensure that all trainees had applied their learning from the course in the workplace, although interview questions extended beyond the context of the course. We designed this study to capture participants’ experiences at various time points to ensure that perceptions reflected a range of experiences with simulation, including both learners’ differing experiences of simulation as they entered the course, their shared experience with simulation during the course, and differing experiences after the course. This was important as multiple interviews improved the potential for students to think *broadly* and *generally* about their perceptions of simulation as a learning modality (this study’s purpose), as opposed to report on only their satisfaction with specific simulation experiences (the focus of most extant literature). The initial interview questions were developed by the research team and revised based on feedback from three pilot interviews. Topics addressed during each interview included trainees’ perceptions of simulation as a clinical learning approach, the perceived value of simulation-based training, which skills are best taught using simulation, characteristics of effective and ineffective simulation-based training they had received, perceived advantages and disadvantages of simulation, and perceived differences between learning in the clinical and simulated setting. Additionally, during the follow-up interviews trainees were asked if the course changed their view of simulation-based training in any way. Each round of interviews was informed by the last in terms of probing more or less on particular topics. Interviews were transcribed verbatim and were organized using NVivo software (QSR International Pty Ltd., Doncaster, Victoria, Australia).

### Data analysis

Data were analyzed using a qualitative descriptive analytic approach informed by constructivist grounded theory techniques to enable us to capture, organize, and (re)present a comprehensive summary of the particular case, a particular simulation-based course. According to Sandelowski, case study scholars and qualitative scholars in medical education,^28^ a number of analytic approaches can inform qualitative description as long as they are epistemologically congruent. In our case study, constructivist grounded theory approaches to coding were helpful and well-aligned; 20,21,23,29,30 therefore, the inductive coding process began with initial coding, in which data from all interviews were labelled according to their topic on a line-by-line basis. This coding was completed individually by three investigators (CMW, AG, and SN). Subsequently, the entire research team met to compare interpretations, review codes, and together create preliminary categorizations, reconciling different viewpoints through discussion with the multidisciplinary team of authors.^31^ The investigators not directly involved in coding used their clinical and research expertise to help clarify and critique the findings. A preliminary set of categories was developed based on the team discussions of initial coding. These categories were then applied for the focused coding stage, in which the preliminary categories organized initial codes. The team revisited transcripts again, expanding and refining the categories. Upon meeting again to review this next round of analysis, five main categories were refined and defined by merging redundant categories and describing key features of each; at this point, two categories were merged into one resulting in four categories (Table 1). These defined categories were then used to return to the data to look for additional clarification and deep description of categories, discrepant data that may suggest the need to change a category or category’s definition, and to identify overlap and linkages between categories. Throughout this process, data were compared within and across transcripts and interview time points, with verbatim quotations reviewed and compared. Finally, the research team met to organize a descriptive categorical framework for the data including relationships between categories, which led to the framework presented in the results section of this paper.

Reflexivity is an important consideration in qualitative research.^32^,^33^ Our research group was comprised of clinicians with experience participating in and supervising simulation-based education, and non-clinician education researchers who brought outsider perspectives, which are useful in qualitative research,^34^ and qualitative research expertise to the data analysis process. The team engaged in dialogue to question and challenge one another’s assumptions throughout analysis. Two of the authors (CMW and SCG) were course faculty; however, they were not involved in the recruitment, consent or data collection process. The interviewer was a medical student uninvolved in the course.

## Results

The analysis included 36 interviews from one first-year gastroenterology resident and 11 second-year surgical residents (mean age 29.5 (range: 27–35); nine males and three females). None of the trainees had prior endoscopy experience; however, they all had previous simulation experience during medical school and residency training, including exposure to standardized patients, mannequins, part-task trainers, and human cadavers. Data analysis yielded four important categories of insights into trainees’ perceptions of simulation-based training within our case study: (1) pragmatic purposes of simulation, (2) simulation as a safe space, (3) optimal design: integration and tension, and (4) perils and pitfalls. Trainees’ perceptions of simulation did not differ across interview time points in that the final categories were consistent across all three interviews. The four categories are explained below with illustrative quotations from the dataset.

### Pragmatic purposes of simulation: Laying the foundation

Trainees were focused on simulation for technical skills training for novice learners. They had a markedly narrow view of simulation’s purposes. Specifically, they tended to see its purpose as limited to early development of foundational techniques and equipment familiarization. They discounted its usefulness in helping them develop non-technical competencies, even when probed specifically about opportunities afforded by simulation to develop skills such as communication.

In terms of familiarization with equipment, residents appreciated being able to arrive at an actual clinical encounter without having to invest time in learning how to utilize equipment in that moment. As one trainee said:

*You’re not wasting the whole time [with] the patient just trying to learn how anything works, either the laparoscope or the colonoscope or anything. So, I think that helps a lot.* (Resident 4, pre-course interview)

Further, residents appreciated the ability to repetitively practice foundational technical skills such as suturing, as illustrated by this statement:

*I practiced it on the simulator hundreds of times and the first time I had the chance to do it in the OR, I was able to load my needle. At least I was able to do the basics: load my needle, get everything aligned like I know I like it and actually do the procedure. At that point I was actually able to follow through with the task. Yes, it was not as smooth as it could have been but I think if I hadn’t practiced on the simulator when I would first do it I would have dropped the needle, I wouldn’t have been able to go through the procedure*. (Resident 5, pre-course interview)

Trainees’ views that simulation was an educational approach for novices were pervasive and ubiquitous. Highlighted by this excerpt, they regarded simulation as a stepping stone in the hierarchy of teaching modalities, not to be revisited:

*I think once you’ve gotten the simulation-based training done then its fine to move on and do the real thing. You’ve got to take the training wheels off the bicycle. Continuously forcing somebody to go back and do more simulation training, I don’t think is helpful.* (Resident 3, follow-up post-course interview)

In response to whether simulation could be helpful for learning more broadly, a resident states his view that simulation served a technical purpose:

*From a technical point of view, you can gain a lot. From any other learning perspective, I don’t really see how it can be of benefit but it just might be the limits of my understanding.* (Resident 3, pre-course interview)

Similarly, trainees tended to discount simulation as a means to train advanced procedures:

*It can be used to teach basic principles. How to throw a stitch, how to tie a knot. But I question how useful they are for performing more complicated procedures.* (Resident 7, pre-course interview)

### Simulation as a safe space: A learner-centered environment

Residents were acutely aware of the negative consequences that could arise from their status as trainees in a clinical setting, where patient care must be prioritized, including limited time for repetitive practice, teaching and feedback and no ability to take risks and try different techniques. Simulation was recognized as providing trainees with a safe learner-centred environment away from the pressures of the clinical setting, which allows them the opportunity to ask questions, learn through exploration and commit mistakes. Trainees recognized the potential consequences of being or appearing uncertain, including losing the patient’s and trainer’s trust, compromising patient safety, and tarnishing the trainee’s confidence and reputation. Trainees saw these consequences as constraints on their clinical learning experience. Therefore, simulation-based training was perceived to provide a means to circumvent these barriers to learning within the clinical context, as reflected by one resident:

*I think when you are doing procedures you tend to ask less questions because patients can hear you… So, I think [simulation] allows you to get through the bumpy start, ask as many questions in a safe environment where you don’t have a patient looking back at you who needs to trust in you for your technical abilities.* (Resident 5, pre-course interview)

Almost universally, trainees suggested that simulation-based training uniquely prioritized learning, a rare opportunity amidst the struggle to optimally balance learning and service. Additionally, it provided a risk-free practice environment that facilitates exploration and problem-solving without negative consequences:

*In a simulated setting, you have the advantage of trying it multiple times and making mistakes and learning from those mistakes. Whereas in reality, with a real patient, with their life in your hands, you can’t do that.* (Resident 12, pre-course interview)

Without simulation experience, the stumbling necessary for learning occurs in the real world and residents recognized the potential negative consequences that this could have on their learning:

*In a clinical setting, if you do it once incorrectly, it is very unlikely for them to allow you try to continue to do it because it will be unsafe for the patient. So you either do it once correctly or you lose your ability to try to troubleshoot and figure out how it should be done.* (Resident 4, pre-course interview)

### Optimal design: Integration and tension

According to trainees, optimal design features for simulation-based education included integration of simulation meaningfully within the overarching curriculum. In particular, they suggested a need for a closer and more deliberate link between skill acquisition within the simulated and clinical context:

*I think [it] is very useful, to be able to, in some close proximity, to learn the skill and then to actually try the skill.* (Resident 11, pre-course interview)

Several residents described struggling to balance perceived educational needs and service demands. They emphasized the importance of implementing supports that enable trainees to take time away from service to engage meaningfully in simulation-based learning:

*In programs where they depend on you to be slumming away and working for them, it looks like you’re not working. It looks like you’re only learning. So that’s sort of the vision of someone who wants to preserve the service model of education, where all of a sudden you’re maybe not putting in time doing work. But you know the counterargument would be obviously that you’re more quickly learning the skills and getting to the end destination, which if it truly is a training program, should be the true goal.* (Resident 2, pre-course interview)

Many trainees articulated the tension they experienced between a desire for appropriate and timely feedback versus independence. They clearly recognized the opportunity afforded by simulation to work through problems in a risk-free setting and appreciated when educators permitted independent problem-solving:

*I think there’s a fine line between sorting it out in your own mind and getting feedback. So I think as an educator it’s very difficult to stand and watch somebody make the same mistake over and over again but I think you do need to make a mistake in order to learn…It’s like you need some of both.* (Resident 12, follow-up post-course interview)

### Perils and pitfalls

Trainees perceived simulation as a learning modality with the potential to misteach or engrain “bad habits,” particularity if the simulation task or activity failed to align closely with reality or if there was a perceived lack of model fidelity. As one resident explained:

*It’s like driving. If you learn to drive in a video game you may turn the car a certain way that doesn’t really work in real life. So it’s the same idea you might learn to do certain things on a simulator... that don’t apply in real life. So you can pick up certain habits that work well here that are contra-beneficial in real life.* (Resident 3, pre-course interview)

Feedback was recognized by trainees as an essential feature to help identify and correct errors to prevent “bad habits” from forming:

*One-on-one training with an expert in whatever you’re simulating on helps. I think when you go and practice on your own, you can practice something that is wrong over and over, and if you don’t have any feedback then you’re not really learning anything. You learn how to use the instruments but you don’t get the extra level of training so I think that’s very specific, you do need the one-onone training to try to fix the mistakes, and then if the simulation, the closer to reality the simulator is, the more you gain out of it.* (Resident 4, pre-course interview)

Trainees also expressed concerns regarding the potential for simulation-based training to foster preconceived expectations, beliefs or assumptions that may act to delimit a residents’ ability to think more broadly within the clinical context:

*The simulator puts you into a certain state of mind and you come in with sort of preconceived notions, some of which are helpful. I don’t want to say it wasn’t helpful. Certainly, some of the aspects were helpful and some were probably counter-productive as well because you get in your mind it’s going to be a certain way and it isn’t. So, you find yourself trapped thinking it should be a certain way and it’s not.* (Resident 3, follow-up post-course interview)

Furthermore, several residents remarked that simulation-based training could potentially instill a false sense of confidence and capability; something which could compromise patient safety if left unchecked:

*If you get too comfortable doing it on the simulator, if you lead yourself to believe that the simulator is the whole of reality, then you’ll go into the real situation and it will probably hurt you to make that assumption.* (Resident 3, immediate post-course interview)

## Discussion

This study is, to our knowledge, the first qualitative exploration, in general, of postgraduate medical trainees’ perspectives on simulation as a learning platform for clinical skills. Residents in this study had a markedly restricted view of simulation as a technical training tool that allows novices to practice foundational skills in a learner-centered environment. This finding conflicts with the extant literature that positions simulation as a training platform with the potential to teach a broad array of skills and levels of trainees though a variety of simulation modalities. For example, simulation has shown utility in teaching basic procedural skills and non-technical competencies, such as collaboration.^4^ In addition, there have been calls from faculty to enhance the use of simulation-based learning for team training, interpersonal skills, and in an interprofessional context.^2^ However, residents in this study consistently perceived simulation-based learning more narrowly despite being primed by course objectives relating to non-technical skills and exposure to simulation-based training targeting non-technical skills both prior to and within the context of the course (e.g. IPPI scenario training with a standardized patient and nurse) and being probed about non-technical learning during the interviews. In line with social cognitive theory, in this discussion we attempt to explain our findings with consideration of personal (including cognitive), behavioural, and environmental dimensions that may impact residents’ perceptions of learning through simulation.

Perhaps the predominance of biomedical knowledge and the pre-eminence of procedural skills in medical curricula,^35^,^36^ produced our participants’ restricted view of the purposes and promises of simulation. The way trainees view simulation as a learning modality is consequential; theories of frame reflection^37^ and problem-framing^38^ suggest that trainees’ restricted view of the purpose of simulation could impose limitations on their learning. How residents frame a learning opportunity may lead them to selectively attend to certain variables, overlooking other possibilities for learning or problem-solving.^37^,^38^ Thus, when the environment of learning has been influenced by the explicit and hidden curricula^36^ to focus predominantly on biomedical knowledge and procedural skills, trainees may be primed to enter a simulation-based learning experience intent on developing technique alone, missing opportunities to learn non-technical competencies.^39^ In our case study, despite exposure to standardized patient-based scenarios^18^ designed to address broader non-technical competencies related to colonoscopy,^24^ and being primed by the course objectives to focus on non-technical skills, such as communication, trainees’ perceptions did not noticeably expand to include non-technical competencies as a particular focus of simulation. In line with social cognitive theory, the introductory nature of the course or the way in which the course objectives were taught during training may have predisposed learners to focus on technical skill acquisition. This narrow view may have impacted trainees’ learning of non-technical skills; unfortunately, learning outcomes were not specifically measured.

Another systemic explanation for trainees’ narrow view of the purpose of simulations could be the way in which simulation-based learning opportunities are developed and integrated within curricula. A number of trainees articulated the need for simulation to be integrated more thoughtfully into overarching curricula, such that simulated training and assessment are integrated with and complementary to other learning events, to truly enhance more traditional forms of education. This sentiment was echoed by simulation program directors and administrators who were interviewed as part of a recent environmental scan on simulation.^2^ In our case study, while non-technical skills were taught, the teaching was not explicitly linked to non-technical skills teaching within the trainees’ wider residency curricula which may have influenced learners’ perceptions. In many countries, postgraduate medical training is moving towards competency-based curricular reforms. In line with this development there has been a paradigm shift, in some places and to some extent, from a time-based to an outcomes-based approach that requires documentation of proficiency.^40^,^41^ Perhaps the explicit recognition and assessment of non-medical expert roles^39^ such as collaborator, communicator, and advocate, that overtly acknowledge and value facets of physician competence in addition to and in conjunction with biomedical expertise, may act to emphasize their importance and lead to a shift in trainees’ perceptions (and values) over time.

Cognitive load theory may also help to explain trainees’ lack of focus on non-technical skills. The limited attentional capacity of humans permits individuals to attend to a finite amount of information simultaneously, thus imposing constraints on capabilities.^42^ The increased amount of cognitive processing required to focus on both technical and non-technical aspects of the procedure may have imposed a high extraneous cognitive load on the novice trainees in this course such that they chose to focus their attention on just the technical aspect of the procedure. Potential solutions to this issue include training non-technical tasks in isolation, providing trainees with more guidance, or ensuring mastery of basic technical skills prior to integration of technical and non-technical skills.

Research has demonstrated that engagement in simulation-based training improves trainee confidence,^46^ and there is evidence to support the presence of cognitive bias towards overconfidence in clinical performance following simulation-based training, which is amplified in those with risk-taking attitudes.^47^,^48^ However, this is the first study to highlight residents’ insight into the potential to develop overconfidence as a result of simulation-based training and the corresponding unintended consequences of overconfidence on patient safety. Overconfidence causes issues from an educational perspective since it provides clear evidence of a breakdown in the learners’ ability to monitor their own learning effectively.^49^,^50^ Additionally, in line with social cognitive theory, alterations in a trainees’ perceived self-efficacy can interfere with performance.^50^,^51^ Research in the context of written knowledge-based multiple choice question tests has shown that judgment bias can be reduced by providing feedback to students regarding both their performance and confidence accuracy on specific questions.^49^ However, potential strategies aimed at preventing overconfidence, such as alterations in feedback delivery or assessing students for self-monitoring capacity,^52^ have not been examined within the context of simulation-based training. Additionally, as mentioned by one trainee in this study, it is important for educators to clearly outline both the purpose and limitations of a simulation activity to help learners gain an appropriate level of confidence, but maintain humility and realistic expectations for the transfer of simulation experience to real-world performance.

An important finding in this work was the emphasis residents placed on the potential for simulation to engrain “bad habits” and foster preconceived expectations that may act to delimit their ability to think more broadly within the clinical context. One-on-one supervision with feedback and case variability were strategies used during the course to help mitigate these effects. Future research could determine whether this concern for learning bad habits is warranted, and what to do to mitigate any perceived or actual risks. Realism of the simulation task and perceived model fidelity were identified by trainees as characteristics essential for transfer to real life. This belief, however, is not supported by the simulation literature which has shown that increases in realism and fidelity are not matched by equivalent gains in performance.^43^–^45^

Our results corroborate the key features of simulation-based teaching that lead to effective learning, as described by Issenberg et al.^1^ in their review of high-fidelity simulation, in the following ways: first, residents appreciated simulation as a training platform that allowed for engagement in repetitive practice, a foundational element of deliberate practice necessary for attaining and maintaining expert performance;^53^ they also felt simulator validity was important and expressed appreciation for curricular integration as a means to foster a direct link between learning in the clinical and simulated environments and to decrease the tension residents experience in trying to balance their educational needs with service demands; finally, trainees clearly valued simulation-based training both as an opportunity for feedback, and paradoxically for independent trial and error.

This study is not without limitations. This case study was conducted at a single academic centre with a small sample of postgraduate trainees from procedurally-focused specialities. Different specialties have shown varied dispositions toward the learning of non-procedural skills,^54^ and the views of residents in procedurally-focused specialties like Gastroenterology and Surgery may differ from learners who are predominately exposed to other forms of simulation, such as multidisciplinary team training targeting crisis resource management skills, or simulation focused primarily on non-technical competencies (e.g., communication skills training with standardized patients). The pervasiveness of the perceptions across our study sample, and our focus on simulation as an educational approach in general (as opposed to trainees’ perceptions of one instance of simulated learning) suggests that our results will likely transfer across contexts; however, further research is warranted. Secondly, because the interviews were conducted within the context of an endoscopy simulation-based course, the heavily technical nature of the skill may have precluded trainees from thinking broadly about simulation as a clinical learning approach, thus discounting its value for teaching non-technical skills. To help mitigate this potential effect, the first interview was conducted prior to the course and trainees were asked, in all interviews, to think broadly about their experiences with simulation (not just in the context of the course). Additionally, the course objectives did encompass non-technical skills, including communication, and situational awareness. The study, however, was limited in that we did not conduct concurrent observations which may have helped to further elucidate the true balance of teaching and feedback regarding technical and non-technical skills during the course. Finally, we were unable to carry out a member check of the results with study participants to ensure emerging insights from data analysis represented a reasonable account of their experience and intended meaning.^55^ That said, we did interview participants three times over seven weeks, and, interestingly, perspectives of trainees did not change throughout the study timeframe. While our case was situated within one simulation-based course, trainees had differing experiences of simulation as they entered the course and they learned in a variety of ways afterwards. The lack of change in their perspectives toward simulation is thus interesting and it may be worth conducting future research that interviews participants over time and across experiences.

### Conclusions

In summary, trainees’ learning expectations of simulation were narrower than the range of reported benefits outlined in the literature. As trainees are active participants in their education and social cognitive theory purports that their learning is influenced by their goals, attitudes, values, and experience,^15^ this misalignment can create educational asymmetry that can act to decrease the effectiveness of simulation-based training. Based on these findings, and theories of frame reflection and problem-framing, we suggest that educators must critically attend to the way they frame the purposes of simulation to learners as trainees’ *a priori* perceptions of simulation may delimit the focus of their learning experiences in that learners may focus their time and energy on tasks they view as “high-value” while neglecting others.^56^ Further research, necessary to determine the transferability of our findings to other institutional settings, disciplines and training levels, could greatly enrich our understanding of this phenomenon. Furthermore, we focused on the perceptions of *trainees*; a related knowledge gap exists regarding how clinical *teachers* view simulation. Future exploration of the perspectives of faculty of this learning platform would add valuable empirical data to the simulation-based education knowledge base.

## Figures and Tables

**Table 1:** List of initial codes and final categories*

Initial Codes	Final Categories
Introduction to equipment	Pragmatic purposes of simulation: Laying the foundation
Technical skills/competencies	
Novice or early learning	
Not useful for non-technical skills	
Not useful for advanced procedures	

Ask questions	Simulation as a safe space: A learner-centered environment
Explore freely (techniques, nonjudgmental)	
Learning through and from mistakes	
Foundational skill set	
Provision of basic approach	
Patient safety (away from patients)	
Learner-centered	

Curricular integration	Optimal design: Integration and tension
Structural support to allow for focus on learning versus service	
Balance need for feedback and independence (tension)	
Realism in the design of simulation	

Engraining bad habits	Perils and pitfalls
False sense of confidence	
Doesn’t translate to real life	

* Codes are not listed in any particular order
